# Airway branching has conserved needs for local parasympathetic innervation but not neurotransmission

**DOI:** 10.1186/s12915-014-0092-2

**Published:** 2014-11-11

**Authors:** Danielle V Bower, Hyung-Kook Lee, Rusty Lansford, Kai Zinn, David Warburton, Scott E Fraser, Edwin C Jesudason

**Affiliations:** Division of Biological Sciences, California Institute of Technology, Pasadena, USA; The Saban Research Institute, Children’s Hospital Los Angeles, Los Angeles, USA; Biological Sciences and Biomedical Engineering, University of Southern California, Los Angeles, USA; Division of Child Health, University of Liverpool, Liverpool, UK; Biological Imaging Center, California Institute of Technology, 1200 E. California Blvd, MC 139-74, Pasadena, CA 91125 USA

**Keywords:** Airway branching, Lung morphogenesis, Lung development, Tracheal branching, Trachea development, Innervation, Denervation, Neurotransmission, Parasympathetic, Laser ablation

## Abstract

**Background:**

Parasympathetic signaling has been inferred to regulate epithelial branching as well as organ regeneration and tumor development. However, the relative contribution of local nerve contact versus secreted signals remains unclear. Here, we show a conserved (vertebrates to invertebrates) requirement for intact local nerves in airway branching, persisting even when cholinergic neurotransmission is blocked.

**Results:**

In the vertebrate lung, deleting enhanced green fluorescent protein (eGFP)-labeled intrinsic neurons using a two-photon laser leaves adjacent cells intact, but abolishes branching. Branching is unaffected by similar laser power delivered to the immediately adjacent non-neural mesodermal tissue, by blocking cholinergic receptors or by blocking synaptic transmission with botulinum toxin A. Because adjacent vasculature and epithelial proliferation also contribute to branching in the vertebrate lung, the direct dependence on nerves for airway branching was tested by deleting neurons in *Drosophila* embryos. A specific deletion of neurons in the *Drosophila* embryo by driving cell-autonomous RicinA under the pan-neuronal *elav* enhancer perturbed *Drosophila* airway development. This system confirmed that even in the absence of a vasculature or epithelial proliferation, airway branching is still disrupted by neural lesioning.

**Conclusions:**

Together, this shows that airway morphogenesis requires local innervation in vertebrates and invertebrates, yet neurotransmission is dispensable. The need for innervation persists in the fly, wherein adjacent vasculature and epithelial proliferation are absent. Our novel, targeted laser ablation technique permitted the local function of parasympathetic innervation to be distinguished from neurotransmission.

**Electronic supplementary material:**

The online version of this article (doi:10.1186/s12915-014-0092-2) contains supplementary material, which is available to authorized users.

## Background

Parasympathetic (cholinergic) signaling is reported to regulate morphogenesis and organ regeneration via effects on epithelial stem cells [1,2]. Similarly, autonomic signaling has been observed to regulate epithelial tumour growth and spread, as well as progenitor responses to organ injury [3,4]. Therefore, the neurogenic regulation of tissue biology, and epithelia in particular, appears as important to understand as the angiogenic regulation of cancer.

To date, these wide-reaching roles of neural regulation of epithelial biology have been attributed chiefly to neurotransmitter signaling. However, it is unclear whether the nerves serve other purposes, such as provision of important structural cues and secreted factors. Moreover, neural effects on epithelial behaviour may be mediated indirectly, via an adjacent vasculature, for example. Vascular interactions influence endodermal development in the lung [5]. Distinguishing a role for direct interactions between nerves and epithelium versus neurotransmitter-mediated signaling or intermediary guidance via the vasculature is important in understanding the mechanism of neural regulation of epithelial tissue morphogenesis.

Pharmacological blockade has provided a relatively simple means to delineate the role of neurotransmission. However, studies have struggled thus far to distinguish other possible roles of innervation. One method that has been tried in order to explore the local roles of innervation has been to surgically dissect out nerves from organs [1]. However, the physical disruptions associated with the surgical technique risk incurring major artefactual changes in the epithelium and indeed other cell lineages. Furthermore, gross dissection disrupts the intimate relationships between nerves and vasculature, confounding whether neural effects on epithelia are direct or mediated indirectly via the vasculature.

Here, we sought to address these problems, first by developing a new laser ablation technique to provide highly selective targeting of intrinsic neurons within the developing lung. Lung explants branch in culture. When the nerve cell bodies lie outside the lung, namely sympathetic and sensory neurons whose cell bodies reside in centralised ganglia, their axons degenerate after lung explantation from the embryo. However, cholinergic nerves whose cell bodies lie within the lung maintain their axon extensions through the lung. These intrinsic neurons have been reported to play key roles in the morphogenesis of glandular epithelia [1]. Therefore, our new technique seeks to interrogate the role of these intrinsic neurons in a major epithelial organ, the lung, while avoiding the need for surgical disruption of that organ.

Our second approach was to eliminate the vasculature as an intermediate variable, by testing the role of innervation in airway morphogenesis in *Drosophila* embryos. This is also attractive because the genetic regulation of both airway morphogenesis and neurogenesis is conserved between flies and mammals, allowing us to test for a conserved role for neural guidance of airway branching. Expression of the RicinA toxin is confined to the developing nerves using a specific promoter, safe in the knowledge that this cytotoxic A subunit of the toxin, even if liberated from dead cells, cannot enter bystander cells because the B subunit is absent. Using *Drosophila* embryos for comparative studies has further benefits because once the tracheoblasts are elaborated in the placodes, airway morphogenesis proceeds by cell migration and does not require further cell proliferation. So, if neural control of airway growth is primarily via provision of mitogenic growth factors, one might expect airway morphogenesis to be independent of neural input in this simpler invertebrate system. Conversely, if innervation is important for epithelial guidance and migration independently of any effect on proliferation, then one would expect airway morphogenesis to depend on nerves in both vertebrates and invertebrates.

Combining these complementary strategies with more traditional approaches using neurotransmitter blockade facilitates a deeper evaluation of how nerves regulate airway growth.

## Results

The lung develops under the control of FGF10-FGFR2IIIb signaling, just like the salivary and prostate glands, in which neural regulation of epithelia has been identified [6,7]. A model of lung development was selected in which neural development is visible without the need for antibody staining. The microtubule-associated protein tau eGFP (MAPT) knockin mouse line has enhanced green fluorescent protein (eGFP) inserted in the tau protein locus. This labels all neuronal cell bodies and axons with eGFP [8]. Hence, neural structures can be seen and followed over time in lung explants.

**Figure 1:**
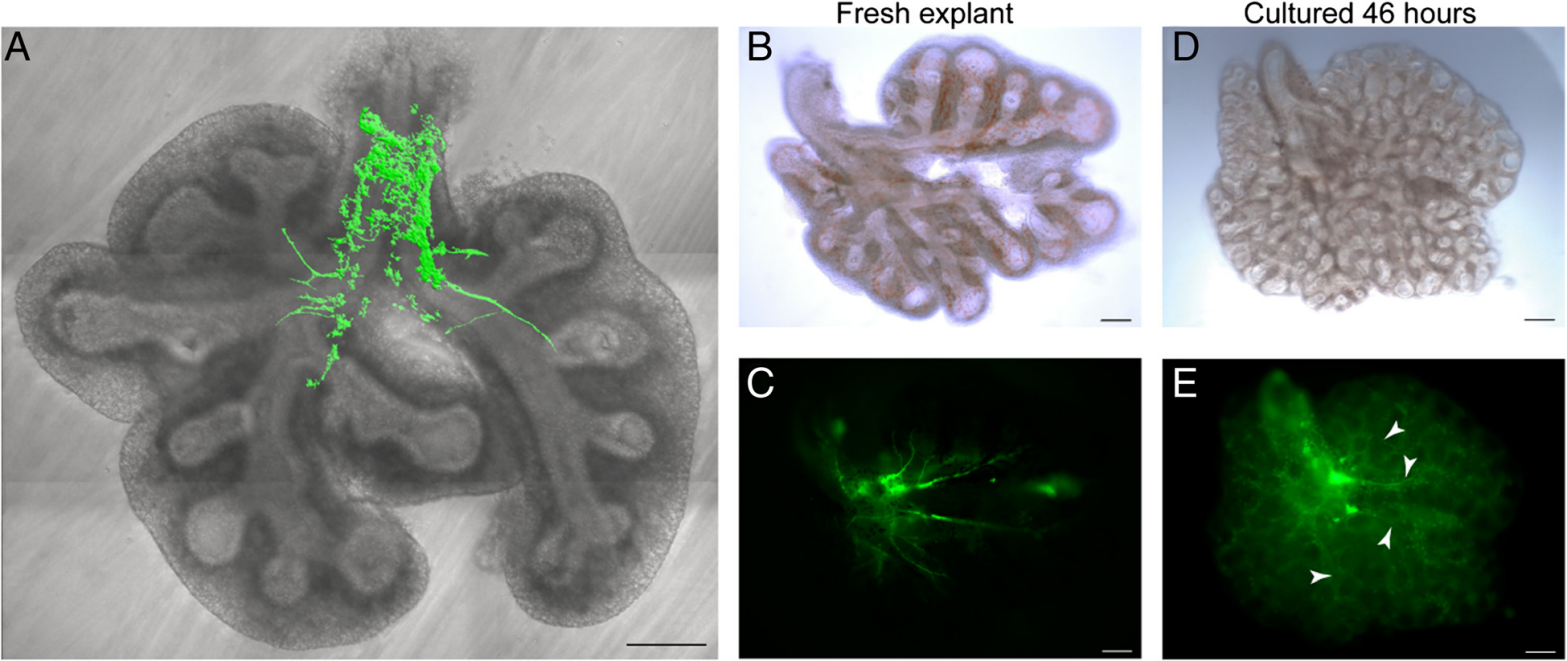
**Lung explants feature epithelial branching with neuronal arborisation. (A)** Axons are visible projecting to each lobe in live lung explants from the microtubule-associated protein tau eGFP (MAPT) mouse line. The vagus nerve has been removed for clarity. Lungs were cultured to assess if nerves continue to arborise with the epithelium following explantation. A fresh explant is seen in brightfield **(B)** and epifluorescence **(C)** to visualise the nerves. After 46 hours in culture, the same lung has branched considerably (**D**, brightfield). **(E)** By epifluorescence, the nerves are still present with axons projecting along the epithelial branches (examples indicated by white arrowheads). Scale bars = 200 μm. eGFP, enhanced green fluorescent protein.

We found that epithelial budding and neural arborisation proceed in tandem over time in culture (Figure 1; n >15) as they do *in situ* [9]. This arborisation arises from post-synaptic parasympathetic neurons whose cell bodies reside within the lung and which survive explantation. Indeed, they continue to elaborate nerve axons along the epithelial tubes as airway branching proceeds. In contrast, sympathetic or sensory neurons do not persist in lung explants as their cell bodies lie outside the lung.

**Figure 2:**
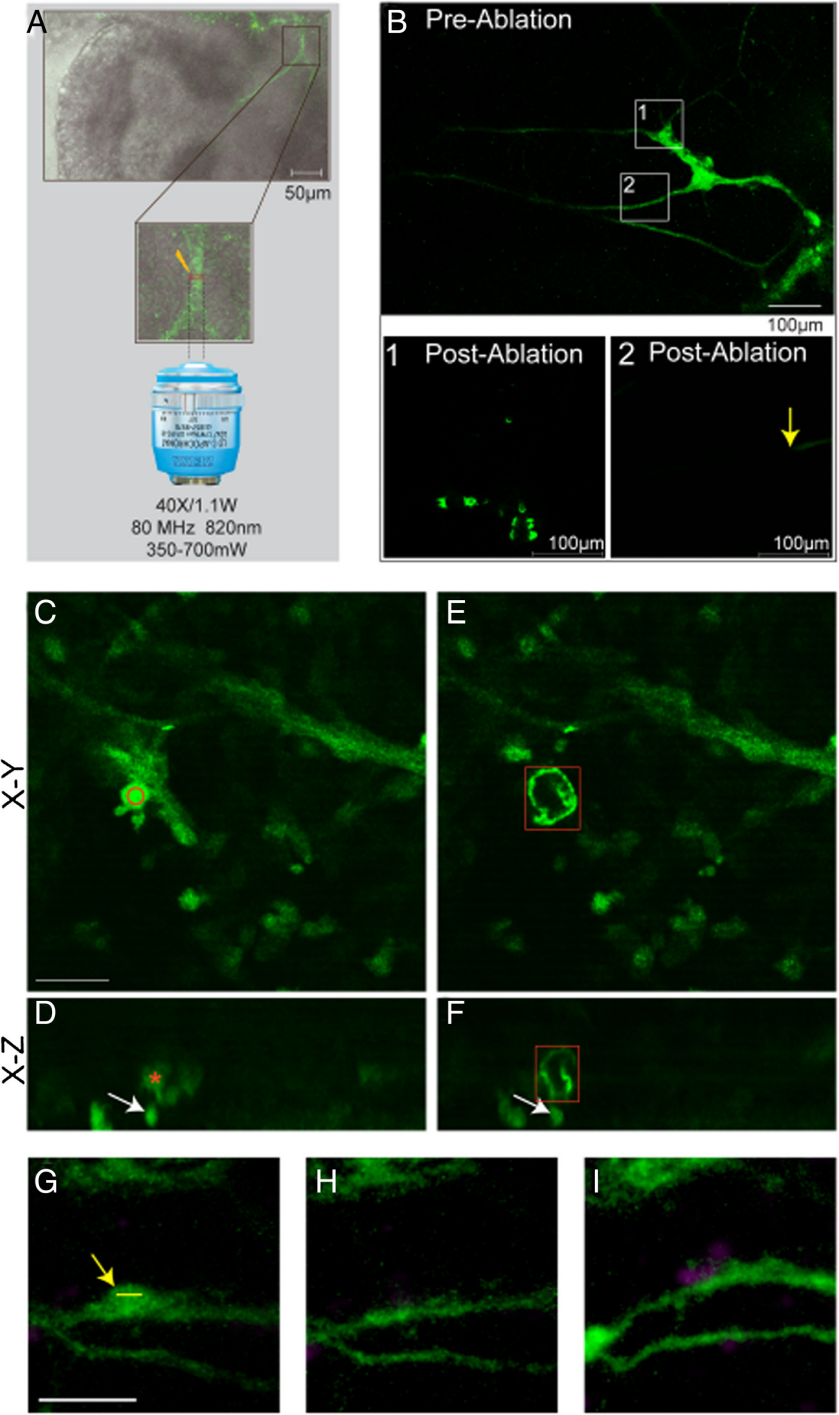
**High-energy two-photon laser targeting eliminates neural cells. (A)** Neurons were identified in MAPT lungs by eGFP fluorescence with confocal microscopy. Infrared light (820 nm) was focused to target cell bodies or axons. **(B)** Neurons were imaged pre- and post-ablation. Cells or axons in boxed regions (top) were targeted and are seen at higher magnification post-ablation. (B1) After targeting cell bodies, a ring of autofluorescence is seen, or the cell is no longer visible. (B2) After slicing axons, eGFP is dissipated distal to the cuts (yellow arrow). *Targeting a cell confines ablation to that cell:* **(C)** Pre-ablation, a neuron of interest is shown in the X-Y plane with the red circle indicating the ablation focus. **(D)** The same neuron is shown in the axial plane (red star), with a neuron directly behind it (white arrow). **(E)** Post-ablation, a ring of autofluorescence marks the destroyed cell (red box). **(F)** Viewed axially, the ablated cell (red) is seen with the unaffected neuron behind it (white arrow). Scale bar = 20 μm throughout. *Bystander cell death was assessed post-ablation in MAPT lung explants treated with Topro3 dead cell indicator:* **(G)** A pre-ablation projected image stack shows cells and axons, with the yellow arrow pointing to future targets and the yellow line to the region of laser focus. **(H)** A post-ablation image stack was collected immediately following ablation to show that targeted cells are no longer visible. **(I)** After four hours in culture, the explant was re-treated with Topro3 and the same region imaged. The few targeted cells are permeable to indicator at the ablation site (purple). Scale bar = 30 μm throughout. eGFP, enhanced green fluorescent protein; MAPT, microtubule-associated protein tau eGFP.

Laser ablation of intrinsic pulmonary neurons was developed to test their local roles during morphogenesis, and to do so with single-cell precision, which contrasts with alternative methods, such as wholesale ganglion excision or pharmacological blockade [1,2]. Two-photon laser-scanning microscopy provided an efficient and three-dimensionally localised ablation of eGFP-labeled neural cell bodies and axons. The energy from the laser is confined to a volume of approximately 1 μm^3^, permitting focal destruction of individual cells and axons (Figure 2A-B). Hence, lasing a single cell with two-photon energy destroys the target cell, but without imparting any collateral damage to cells immediately adjacent to, in front of, or behind the target (Figure 2C-F). Confocal imaging confirmed that no acute damage is done to bystander cells, including adjacent epithelium, endothelium, nerves or mesenchyme.

Subacute damage to surrounding cells was sought using Topro3-iodide as a dead-cell indicator dye. Even hours after targeted two-photon laser ablation, there were no delayed effects on adjacent bystander cells (Figure 2G-I), reaffirming that the technique provides ablation with single-cell precision. In contrast to prior approaches, such as wholesale ganglion excision, this selective neural ablation avoids gross disruption of organ anatomy, which has major effects on non-neural bystander cells and requires reconstitution of the disrupted explant.

**Figure 3:**
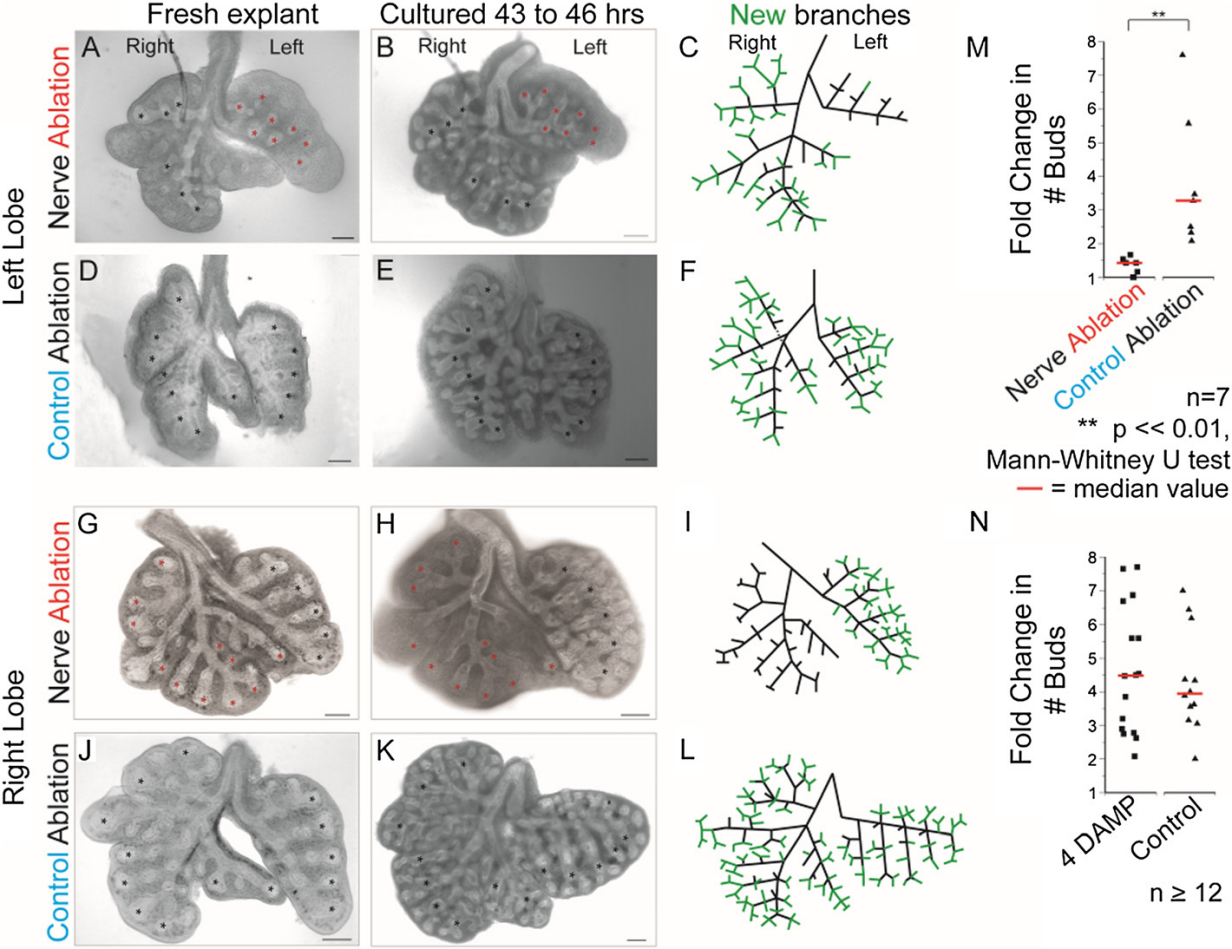
**Nerve ablation halts lung branching** ***.*** **(A)** Nerves were ablated to the left lung but not the right. Red and black asterisks mark left and right lung terminal buds, respectively, at the time of ablation and mark these original positions after 43 to 46 hours in culture in **(B)**. New branches have formed beyond asterisks in the right lung but on the left only one branch has extended slightly. **(C)** The panel diagrams the branches present on explantation (black) and new branches formed over two days (green). **(D)** As a control, equivalent laser energy was delivered to ablate non-neural mesenchymal cells from the left lung of a matched explant, avoiding epithelium, neurons and endothelium. Asterisks mark the original terminal buds and **(E)** shows how budding has progressed beyond these original terminal locations after two days. Both lungs branch substantially, as illustrated in the schematic **(F)**. **(G-L)** shows that neural ablation **(G)** halts right lung branching without affecting the non-intervened left lung **(H,I)** while control ablation **(J)** leaves branching unperturbed in both lungs **(K,L)**. Scale bar = 200 μm throughout. **(M)** Nerve ablation significantly reduces lung branching after two days in culture, while control mesenchymal ablation has no affect. **(N)** Muscarinic inhibitor 4-DAMP did not alter lung budding compared to controls. Red lines indicate median values. 4-DAMP, 1,1-dimethyl-4-diphenylacetoxypiperidinium iodide.

To determine the effects of denervation on epithelial budding, this precise two-photon laser ablation technique was applied to one lung of each explanted pair. Confocal microscopy identified the eGFP labeled neuronal cell bodies and axons within the cultured left lung. At this stage, the explants are fractions of a millimeter in thickness, allowing the complete innervation of the lung to be visualised. Each neuronal cell body was individually destroyed by laser ablation. Likewise, each axon bundle was severed using laser ablation at the level of the proximal airway branches within the left lung. Thus, selective denervation of the left lung was achieved to determine its effects on epithelial budding. A non-ablated control for this intervention was to culture the paired right lung without any laser ablation (Figure 3A-C). A control ablation was performed in a stage-matched left lung explanted from a sibling embryo by imparting identical laser irradiation but applying it to non-neural cells in the mesoderm immediately adjacent to the neuronal cell bodies and axons (Figure 3D-F). Just like target regions for the neuronal ablation, these control ablations avoided the epithelium and endothelium. These nerve-ablated and control ablated explants and their partner right lungs were then cultured for two days.

The laser-denervated left lung added few if any new buds (Figure 3A-C). In contrast, the right lung without any intervention (non-ablated control) developed new buds as usual (n = 7 per group). Laser ablation of non-neural mesodermal cells in the left lung (control ablation) saw the left lung bud as profusely as unperturbed explants (Figure 3D-F). Likewise, budding halted after laser denervation of the right lung, but proceeded normally in the non-intervened left lung (Figure 3G-I) or when laser ablation was targeted only at non-neural mesodermal cells (Figure 3J-L). Overall, bud numbers more than tripled after control ablations, while they flat-lined after neural ablation (Figure 3M). Therefore, it is selective neural ablation rather than any non-specific effect of laser irradiation that abrogates local epithelial lung budding.

Previous studies reported that parasympathetic cholinergic signaling regulated salivary gland budding and epithelial stem cells [1,2]. To test if neural ablation stopped lung budding due to interruption of parasympathetic signaling, lung explants were cultured with and without the muscarinic antagonist 1,1-dimethyl-4-diphenylacetoxypiperidinium iodide (4-DAMP, 10 to 20 M; n ≥12 per treatment). In contrast to reports in the salivary gland, muscarinic blockade (M3) did not affect lung budding at embryonic day 12.5 to 13 when early branching is under way (Figure 3N and Additional file 1: Figure S1 A-D). Similarly, pan-cholinergic blockade with atropine (20 μM, 40 μM; n ≥8 per treatment) failed to alter budding (see Additional file 1: Figure S1E). Even when synaptic release was blocked using botulinum toxin (50 ng/mL, 100 ng/mL, 200 ng/mL; n ≥12 per treatment), lung explants budded normally in culture (see Additional file 1: Figure S1F-J). Together, this shows that the failure of local lung budding after selective pulmonary denervation cannot be attributed to a loss of cholinergic neurotransmitter signaling.

**Figure 4:**
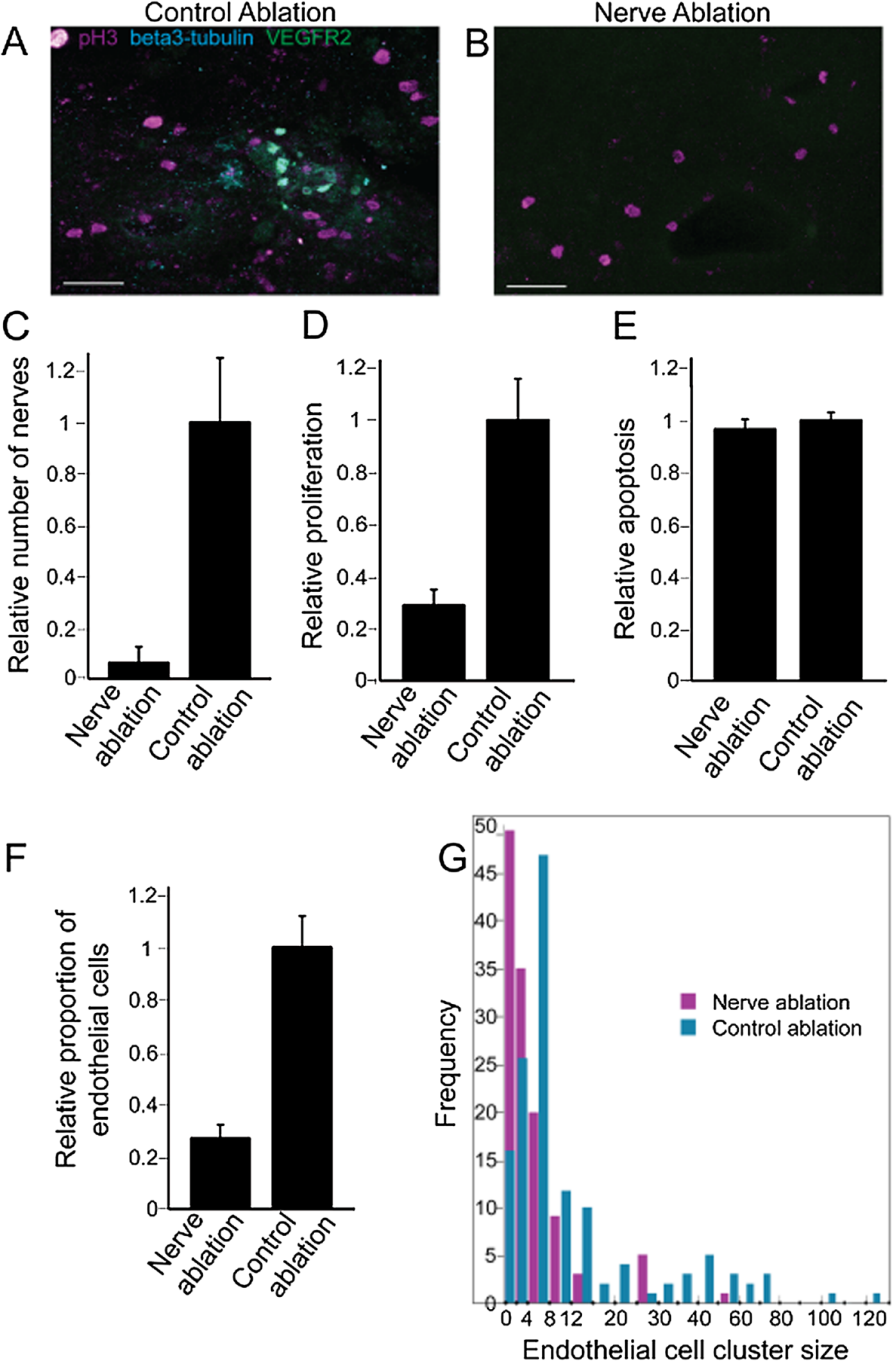
**Laser ablation denervates the lung, resulting in fewer endothelial cells and reduced proliferation.** Sections from control ablated lung **(A)** and nerve ablated lung **(B)** were stained for phosphohistone H3 (pink), neural β3-tubulin (cyan), and VEGFR2 (green). Neural ablation reduces neural cells to 6% **(C)** and cell proliferation to about 30% **(D)** of control ablated samples but with no difference in apoptosis **(E)** measured by cleaved caspase 3 staining (mean, SEM). Compared to control ablations, neural ablation reduces endothelial cell numbers by two-thirds **(F)** and reduces their cluster size **(G)**. Scale bars = 50 μm. Error bars represent S.E.M. For all plots, n = 3 samples, with 10 or more sections analysed per treatment. SEM, standard error of the mean; VEGFR2, vascular epithelial growth factor receptor 2.

Immunohistochemistry on lung explants confirmed that the laser ablation achieved selective neural destruction and also established the effects of denervation on pulmonary cell fates (Figure 4). The number of neural cells and endothelial cells were identified by beta3-tubulin and vascular epithelial growth factor receptor 2 (VEGFR2) staining, respectively (Figure 4A and B). The relative number of neural cells per explant was greatly reduced by neural ablation (Figure 4C; for all plots in Figure 4, n = 3 different samples, with 10 or more tissue sections per treatment).

Pulmonary cell proliferation, measured using phosphohistone H3 (pH3) immunostaining, was reduced after laser denervation (Figure 4D), while apoptosis rates as measured by cleaved caspase 3 immunohistochemistry were unchanged (mean, SEM; Figure 4E). Following neural ablation, VEGFR2 positive vascular endothelial cells numbered about a third of that of normal explants (Figure 4F), and the numbers of cells in endothelial cell clusters were reduced, indicating reduced endothelial proliferation following neural depletion (Figure 4G). Hence, pulmonary neural tissue regulates airway branching as well as epithelial and endothelial proliferation.

**Figure 5:**
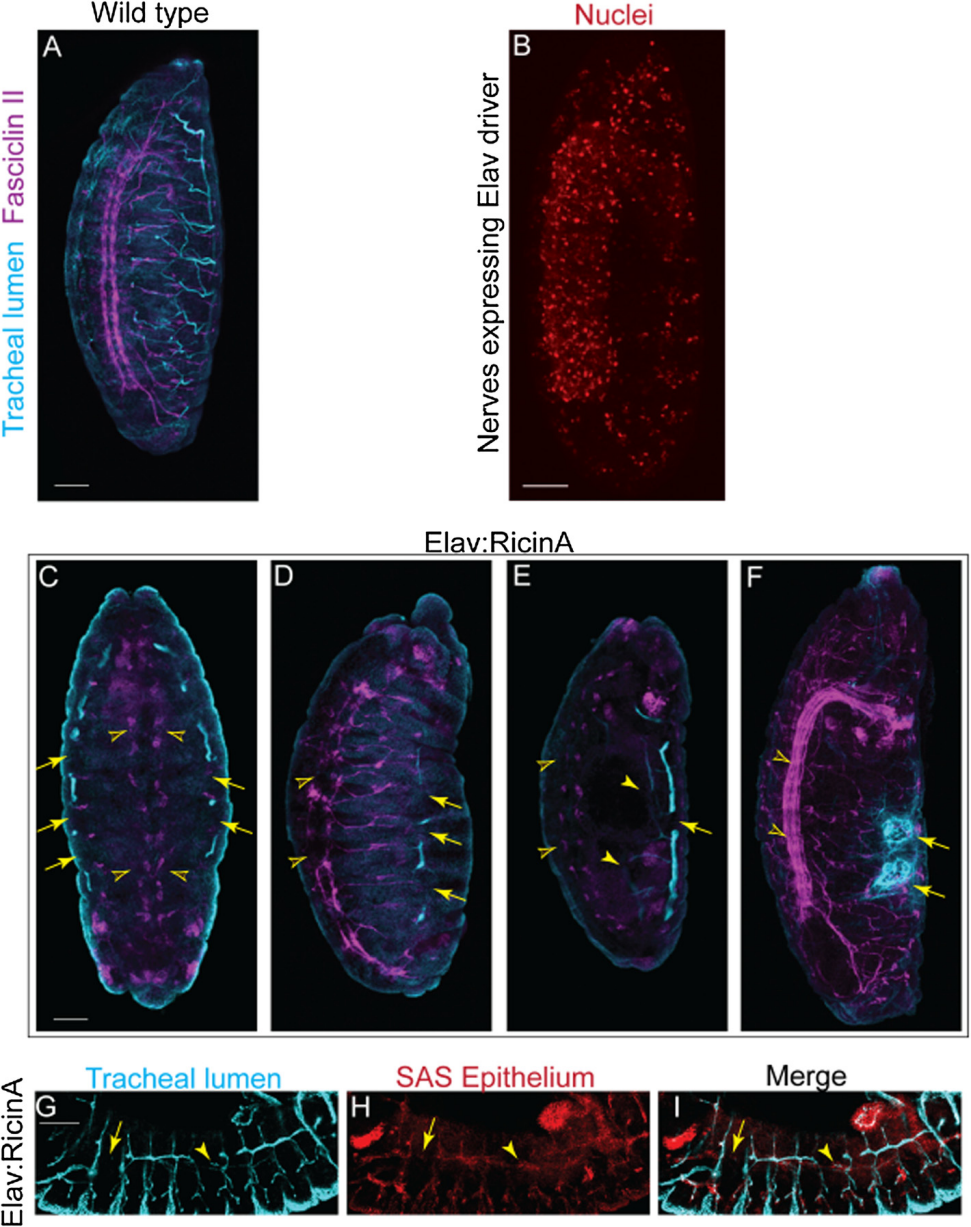
**Innervation is required for** ***Drosophila*** **tracheogenesis.** The RicinA subunit was driven under the pan-neural driver *elav* to lesion neurogenesis in *Drosophila* embryos. FasII staining labels longitudinal tracts of the CNS and some motor axons (magenta). Tracheal structure is stained for lumen protein (cyan). **(A)** shows neural and tracheal structure in wild type embryos. **(B)** Neural nuclei are labeled by activity of the *elav* promoter to visualise the cells affected by RicinA. **(C-F)** show examples of RicinA-mediated neural depletion and its effects on airway development. Longitudinal tracts are often almost eliminated (**C,E** open arrowheads) or occasionally reduced to single disordered and broken tracts (**D**, open arrowheads) or merged with disordered peripheral projections (**F**, open arrowheads). Sections of dorsal tracheal trunk do form but show breaks (**C-E**, arrows). In **(C)**, the tracheal cells that appear to be forming segments of a dorsal trunk are seen improperly on the ventral side of the embryo. Smaller tubes are largely absent by lumen staining, and those that remain are incomplete (**E**, closed arrowheads). In **(F)**, the tracheal cells form severely disorganised balls of lumenised tube (arrows). **(G-I)** show labeling of tracheal lumen **(G)** and tracheal epithelium **(H)** with the merged image shown in **(I)** to test if the discontinuous tubule staining simply represents a lumenisation problem or if there are true gaps in the epithelial tubes. Sections of stained lumen **(G)** are surrounded by epithelial cells **(H,I)** (example indicated by arrowheads). Where gaps in the lumen exist, epithelial cells are absent (**G-I**, arrow). Scale bars =50 μm. CNS, central nervous system.

Next, we sought to determine to what extent neural regulation of airway morphogenesis is separable from neural regulation of epithelial and endothelial proliferation. In the mammalian model, this is not readily testable because epithelial and endothelial proliferation are themselves fundamental requirements for normal airway morphogenesis. Instead, airway morphogenesis was examined in *Drosophila* embryos (Figure 5A) because fly tracheogenesis shares genetic homologies with mammalian lung development but is completed via epithelial migration without an adjacent vasculature or epithelial proliferation (once the requisite number of tracheoblasts has been produced) [10-14].

Selective ablation of nerves in *Drosophila* embryos, therefore, tests whether neural control of airway morphogenesis is conserved between vertebrates and invertebrates and whether neural regulation persists independently of endothelial or epithelial proliferation [15]. Starting at stage 12, the *elav* driver is expressed in developing neural tissue in *Drosophila* embryos (Figure 5B) [16]. Using this driver, we selectively expressed the RicinA toxin in the nervous system (n >30). RicinA is a potent intracellular toxin that kills cells that express it. Lacking a B subunit, it cannot enter bystander cells to kill them [17]. In this model, toxin expression starts to kill neural cells after completion of tracheoblast proliferation but as tracheoblasts are beginning their migration to elaborate the tracheal airways. Therefore, any observed disruptions of the tracheal system derive from a requirement for nerves for the proper migration of tracheoblasts and the structuring of the tubular segments.

Tracheoblast migration is regulated by mitogen signaling and topographical guidance [12,18]. Dorsal trunks form early, while the smaller tracheal branches develop later. Immunohistochemical labeling of the trachea and nervous system showed that selective RicinA-mediated lesioning of neurogenesis led to substantial disruption of airway morphogenesis (Figure 5C-F). Otherwise, the embryos looked grossly normal with appropriate formation of other organs such as the gut. Most often, dorsal trunks were relatively spared but were discontinuous and transposed ventral to their usual location. In contrast, smaller branches including the transverse connectives, lateral trunk, visceral, ganglionic and dorsal branches were smaller or absent. In severe examples, tracheal structure was lost, with the tracheoblasts clumped in spheres (Figure 5F). This suggests that as more neurons are killed by expressing RicinA, tracheoblast migration and segmental fusion are progressively impaired, due in part to loss of neuronal guidance. This explains the relative preservation of sections of a dorsal trunk structure, albeit often displaced from its expected location within the embryo, with more marked loss of the smaller branches that form later. Our tracheal phenotypes cannot be explained solely by indirect effects on other tissues, such as muscle, because they are distinct from phenotypes reported when these non-neuronal lineages are ablated [19,20].

Immunostaining for the epithelium matched that of the 2A12 tracheal lumen marker confirming that the neural ablations cause true disruptions of tracheal structure rather than just perturbed levels of the lumen protein (Figure 5G-I). Together, these mammalian and *Drosophila* studies show that neural control of airway morphogenesis is conserved between mammals and invertebrates and that impacts on airway branching are independent of neural regulation of endothelial cell behaviors.

## Discussion

Innervation has been accorded vital roles in organ development, homeostasis, regeneration and tumourigenesis [1-4]. Much of the evidence for this has accrued from studying the effects of neurotransmitter blockade, so emphasis on the role of neurotransmitters in epithelial regulation is unsurprising.

Avoiding the off-target effects of wholesale ganglion excision, we developed a novel technique for highly precise laser denervation of the lung to show that this halts branching and reduces both epithelial and endothelial proliferation, but in a manner that cannot be reproduced by parasympathetic neurotransmitter blockade. Indeed, contrary to reports in the salivary gland, we found that neural regulation of airway morphogenesis is not mediated by cholinergic neurotransmitter discharge at this phase of development during the early ramification of the bronchial tree. In fact, epithelial lung branching proceeds normally despite anti-muscarinic, anti-cholinergic and, even, botulinum-mediated blockade of neurotransmitter release.

**Figure 6:**
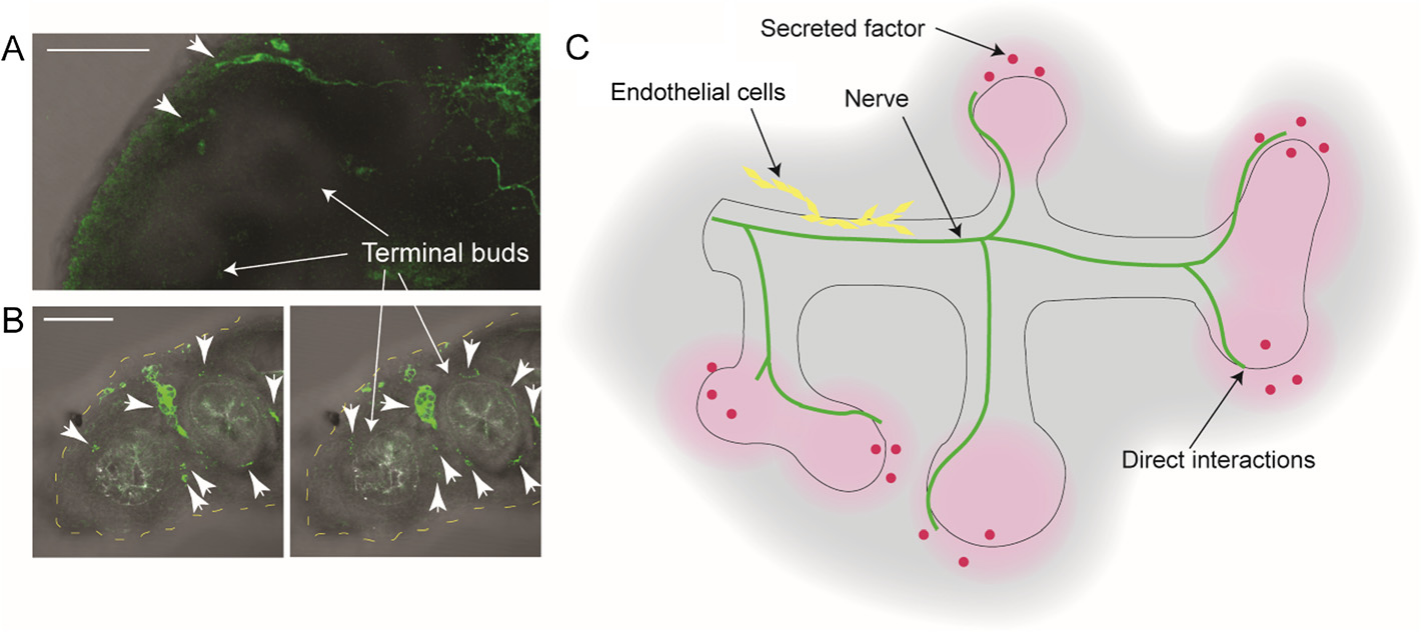
**Pulmonary nerves extend to the budding epithelial tips and may regulate airway branching via secreted growth factors, structural cues, or cellular interactions. (A)** In wholemount cultured explants, nerve fibres are visible reaching peripheral epithelium (anti-β3-tubulin immunostaining, green, indicated by arrowheads). In tissue sections **(B)**, tiny nerve fibres can be seen bordering terminal airway buds. The images are individual adjacent slices of a z-stack obtained with confocal microscopy of sectioned explants. The terminal border of the explant is outlined with yellow dashes for clarity. Scale bars = 100 μm. **(C)** Diagram of possible mechanisms by which nerves may regulate airway branching, including via secreted growth factors, direct cellular interactions or intermediary interactions with other tissues.

This suggests that neural regulation of airway morphogenesis relies on factors other than neurotransmission. Individual nerves can be observed extending to or very near the terminal airway buds (Figure 6A, B), which is consistent with findings reported in other studies [21]. Given the proximity of nerve terminals to nascent epithelial buds, neural impacts on airway branching may be mediated via secreted growth factors, structural cues provided by the nerves themselves, or signaling directly between neurons and epithelium or augmented by an intermediary tissue, such as the vasculature (Figure 6C). Comparison with culturing severed bud tips raises the question of whether neural guidance of branching may be implemented through more than one means of signaling activity. Severed lung bud tips continue to sprout buds in a non-stereotyped manner in culture media, whereas denervation in whole lung explants halts branching entirely. Thus, cultured severed tips may be freed from certain growth constraints, retaining the ability to randomly sprout buds in the absence of innervation, or neurons could play a role, directly or indirectly, in imposing organizational constraints as well as providing stimulatory signaling.

Examination of the plethora of neuronally-secreted factors is beyond the scope of the present study. However, it can be noted that nerve-derived trophic factors would allow adjacent nerves to regulate distal epithelial budding and would explain the reduced epithelial proliferation observed after selective laser denervation.

Selective denervation in *Drosophila* embryos offered a model to examine airway morphogenesis in a context where epithelial and endothelial proliferation does not contribute [22]. Our findings show that neural control of airway morphogenesis is conserved between invertebrates and mammals and persists even where there is need for neither vasculature nor epithelial proliferation.

Clearly, the neural control of airway morphogenesis may still be mediated via disrupting the actions of other unspecified tissues. However, we found grossly normal tissues elsewhere in the *Drosophila* embryos, so there is no strong support for that possibility at this stage. In contrast, a direct effect of the nerves on the tracheal airway is highly plausible in this context, given the close anatomical proximities of neurons and trachea and the dependence of both systems on conserved guidance cues for cell migration.

## Conclusions

We have demonstrated a novel technique employing targeted laser ablation to denervate lung explants without disrupting the structural or cellular integrity of the tissue. Elimination of local parasympathetic nerves abolishes lung branching and diminishes endothelial and epithelial cell proliferation, and these effects are not mediated by neurotransmission. Homologous airway morphogenesis in the invertebrate fly embryo proceeds without tracheal cell proliferation or a vasculature, yet is likewise dependent on local innervation. Taken together, our results argue that nerves have a conserved role in airway morphogenesis, from the fly, where they act without need for proliferation or vascular intermediary, to the mouse, where they have neurotransmitter-independent roles and regulate cell proliferation.

## Methods

### Ethics statement

All experimental protocols were in accordance with the recommendations in the Guide for the Care and Use of Laboratory Animals of the National Institutes of Health. The mouse protocols were approved by the Institutional Animal Care and Use Committee at Children’s Hospital Los Angeles (IACUC protocol #252) and at the California Institute of Technology (IACUC protocol #1401-10G). All mice included in this study were humanely euthanised by exposure to CO_2_ in an inhalation chamber.

### Lung culture, drug treatments and imaging for morphometry

Lungs were explanted from embryonic day (E) 12.5 embryos and cultured at the air-liquid interface on culture inserts as previously described with 50% (D)MEM-50% F12 media plus 2% fetal calf serum (FCS) and 100 U penicillin/100 μg streptomycin (1:100 dilution) [23]. Explants were cultured at 37°C with 5% CO_2_. Fresh media were provided approximately every 24 hours. Lung cultures were treated with the following additions to the media to achieve final concentrations as follows: 4-DAMP (Tocris Biosci 0482, Minneapolis, Minnesota, USA) at 10 or 20 μM, with or without 400 ng/mL mouse recombinant FGF10 (R&D systems 6224-FG-025/CF, Minneapolis, Minnesota, USA) and 50 ng/mL heparin (Sigma Aldrich H3393, St. Louis, Missouri, USA); atropine: 20 or 40 μM (Sigma Aldrich A0132, St. Louis, Missouri, USA); botulinum toxin, serotype A (BTx): 50, 100, or 200 ng/mL (List Biologicals #128A, Campbell, California, USA). BTx was stored at 4°C (in a locked refrigerator), reconstituted at 100 ng/μL in water with 1 mg/mL BSA and handled in a fume hood. Samples were fixed with 4% paraformaldehyde (PFA, Sigma Aldrich, St. Louis, Missouri, USA) to inactivate the toxin prior to imaging, and all contaminated materials were bleached with 10% bleach and/or autoclaved before disposal to inactivate toxin. Materials were disposed of according to Biosafety guidelines. Widefield transilluminated and epifluorescent images of cultured lung explants were captured with an Olympus stereoscope. The number of buds was counted to quantify the extent of branching, and the fold change in number of buds was calculated.

### Laser ablation

Lungs were transferred to coverslip-bottomed imaging dishes. The ablation procedure was limited to one hour per sample, with lungs then returned to culture inserts. Ablations were performed using a long-working distance, high numerical aperture LD C-Apochromat 40X/1.1 water objective for a small focal volume with a digital zoom of 3×. A z-stack was first collected with confocal imaging using 488 nm wavelength light from an argon laser. The same region was then imaged with low intensity 820 nm infrared light with a pulse frequency of 80 MHz from a titanium-sapphire laser. The collimating lens for the infrared light was adjusted until the focal plane was the same as for the 488 nm visible light. A nerve or axon of interest was then identified with confocal imaging, and a small region of interest was drawn within the cell or axon. That region of interest was lased with approximately 350 to 700 mW of average power (30% to 60% power) at 820 nm with a pixel dwell time of 164 μs/px. The area was subsequently imaged with 488 nm light to verify liquidation of the target. The number of scans performed for an ablation was counted, and the same number of scans was performed in a control ablation on another sample. Approximately 100 to 150 individual scans were required to denervate a lung. For the control ablations, mesenchymal cells adjacent to nerves and epithelium were targeted. Epithelium, blood vessels, and nerves were avoided.

### Topro cell death assay

A total of 1 μM of Topro3-iodide (Life Technologies T3605, Grand Island, New York, USA) was added to lung explant media right before targeted ablation was performed and immediately before each subsequent timepoint after culture to visualise dead cells. Topro3 iodide 642 was excited with 633 nm light and was well separated from eGFP emission.

### Antibody staining of lung explants

Lung explants were fixed with 4% crystalline PFA overnight at 4°C then washed four times with phosphate-buffered saline (PBS) to remove the PFA. Lungs were mounted in 3% agarose and vibratome sectioned approximately perpendicular to the length of the explants at a thickness of 75 μm. A vibrational amplitude of 8 and speed of 2.5 were used. Sections were placed in Lab-Tek eight-well chambered slides and blocked for three hours in blocking solution (PBS/0.8% TritonX-100/1% fraction 5 BSA/10% goat serum). Primary antibodies were applied in fresh blocking solution overnight for two to four nights at 4°C with gentle nutation. Primary antibody solution was removed and sections were washed in PBS/0.3% TritonX-100 at room temperature four to six times over approximately four hours and then overnight at 4°C. Secondary antibodies were applied at a 1:500 dilution in blocking solution overnight at 4°C. Secondary antibody solution was removed and sections were washed approximately four times over four hours at room temperature and overnight at 4°C in PBS/0.3% TritonX-100. (See Additional file 2: Table S1 for antibodies used).

To image stained sections, sections were mounted between two coverslips with a small amount of PBS and placed on a copper slide holder for ease of flipping the sample over to image both sides. Confocal and two-photon imaging were performed with a Zeiss LSM 710 microscope with the following collection parameters: 5.5% to 6% 730 nm excitation, 371 to 455 nm collection; 5.8% to 6.0% 488 nm excitation, 500 to 558 nm collection; 0.05% 561 nm excitation, 592 to 631 nm collection; 0.8% to 3% 633 nm excitation, 650 to 746 nm collection. Identical settings were used across samples except when signal was saturating, and then the laser power was adjusted to obtain comparable signal at the surface and depth between samples. The pinhole size for confocal imaging was 4 μm, and the pinhole was open all the way for two-photon imaging. The z-step interval was 4 μm, and the pixel dwell time was 2.55 μs/px. Overlapping tiled z-stacks were stitched using Fiji plugins [24].

### Confocal imaging of live lung explants

Lungs were placed in coverslip-bottomed imaging dishes in a small amount of media and imaged with a Zeiss LSM 510 meta or Zeiss LSM 710 microscope. A 488 nm wavelength from an argon laser was used to image eGFP. To image multiple fluorophores in combinatorial antibody staining, two-photon excitation at 730 nm was used to excite A350 dyes, 488 nm was used for FITC or A488 dyes, 561 nm was used for A568 and A594 dyes, and 633 nm was used to excite A633 and A647 dyes, and bandpasses were set to collect non-overlapping emissions. A Plan-Apochromat 20X/0.8 NA air or LD C-Apochromat 40X/1.1 water objective was used with a pinhole set to 4 to 6.8 μm. Tiled z-stacks of images were collected with a pixel dwell time of 2.55 to 3.22 μs and a z-interval of 2 to 6 μm.

### Statistics

Individual cells were counted in antibody-stained sections to quantify the number of neurons and endothelial cells within lobes of nerve-depleted or control-ablation lungs, and numbers were normalised by the volume of tissue imaged for equivalent comparison between ablations and controls. Ratios of cell numbers were calculated for the treatment group compared to the controls. Means and standard errors of the mean were calculated and plotted using Matlab. Endothelial cell cluster sizes were also plotted using Matlab. Statistical significance in differences in branching of lung explants was determined by the Mann-Whitney *U* test for continuous variables that may or may not have a normal distribution.

### Fly crosses

For pan-neural ablation, the *elav-gal4* driver line was crossed to the *UAS-ricinA/CyO* line. Embryos with neural ablation were clearly distinguishable from normal embryos with no ablation by neural antibody staining. To visualise the cells affected by this ablation, the *elav*-*gal4* driver line was crossed to the *UAS-dsRed* stinger line, which expresses nuclear dsRed in cells expressing the driving promoter. Embryos were collected on grape jelly plates in egg laying chambers and kept moist until they had developed to the stage of interest. For the *UAS-dsRed* cross, embryos were heat shocked for two hours at 29°C five hours before imaging to activate the nuclear dsRed expression, and then they were imaged live three hours later.

### *Drosophila* embryo fixation

For whole mount preparation, when embryos reached stages 13 to 16, they were transferred to glass vials and treated with heptane and 5% PFA in PBS for 15 minutes at room temperature to fix. The PFA was removed and 100% methanol was added, and the embryos were shaken to remove the vitelline membrane. The heptane was aspirated and the embryos were incrementally rehydrated through 70%, 50%, 30%, 0% methanol in PBS and then transferred to PBT (PBS +0.05% TritonX-100 + 0.1% BSA).

### *Drosophila* embryo antibody staining

For antibody staining, embryos were blocked for one hour at room temperature in 5% normal goat serum (NGS) then incubated with primary antibodies in PBT +2% NGS overnight at 4°C. Embryos were washed six times for 30 minutes at room temperature in PBT then blocked for 20 minutes in 5% NGS. Secondary antibodies (Invitrogen, Grand Island, New York, USA) were used at 1:500 in PBT +2% NGS overnight at 4°C. Embryos were washed six times for 30 minutes at room temperature and transferred to PBT/14% glycerol.

### Imaging of *Drosophila* embryos

Whole mount *Drosophila* embryos were arranged on glass slides with permafluor (for fixed embryos) or PBS (for live nuclear dsRed labeled embryos) and coverslipped for imaging. Coverslipped fillet preparations were imaged the same way as whole mount embryos. Confocal and two-photon tiled z-stacks were collected with a Zeiss LSM 510 meta or Zeiss LSM 710 microscope using a 2 μm pinhole (for confocal) and 1.5 μm z-step interval. Appropriate excitation wavelengths and collection bandpasses were selected as above for the fluorophores used. Images were assembled using Fiji stitching plugins [24] and viewed in three-dimensions using Imaris software (Bitplane).

## Additional files

Additional file 1: Figure S1. Unlike the submandibular gland, inhibition of cholinergic receptors does not affect lung branching. Explants were treated with or without the irreversible muscarinic inhibitor 4-DAMP, alone or in combination with branching mediators heparin and FGF10. Freshly explanted lungs treated with 4-DAMP (A) or without inhibitor (B) are shown. The same lungs after 46 hours in culture are shown following culture with 4-DAMP (C) or without (D). At no concentration of 4-DAMP was an inhibitory effect on branching measured. The lung in (C) has more branches than the one in (D) owing to its slightly greater maturity upon explant despite coming from sibling embryos of the same pregnant mother. The branching is proportional to their starting maturity. Scale bars = 200 μm. (E) shows the results of the same experiments repeated with atropine rather than 4-DAMP. Again, neither dose of atropine impaired budding. (F-J) Lungs were cultured with botulinum toxin A starting at (F) and concluding at (H); or without the toxin, starting at (G) and seen after culture at (I). The persistence of normal budding with botulinum toxin is seen in the graph (J). Additional file 2: Table S1. Antibodies used for mammalian lung or *Drosophila* studies.

## Abbreviations

BSA: bovine serum albumin
BTx: botulinum toxin
(D)MEM: (Dulbecco’s) modified Eagle’s medium
eGFP: enhanced green fluorescent protein
MAPT: microtubule-associated protein tau eGFP
NGS: normal goat serum
PBS: phosphate-buffered saline
PBT: PBS plus Triton X
PFA: paraformaldehyde
VEGFR2: vascular epithelial growth factor receptor 2
