# Supplementary figures and images for: Airway branching has conserved needs for local parasympathetic innervation but not neurotransmission
Source: BMC Biol. 2014 Nov 11;12:92. doi: 10.1186/s12915-014-0092-2 (PMC4255442; doi:10.1186/s12915-014-0092-2)

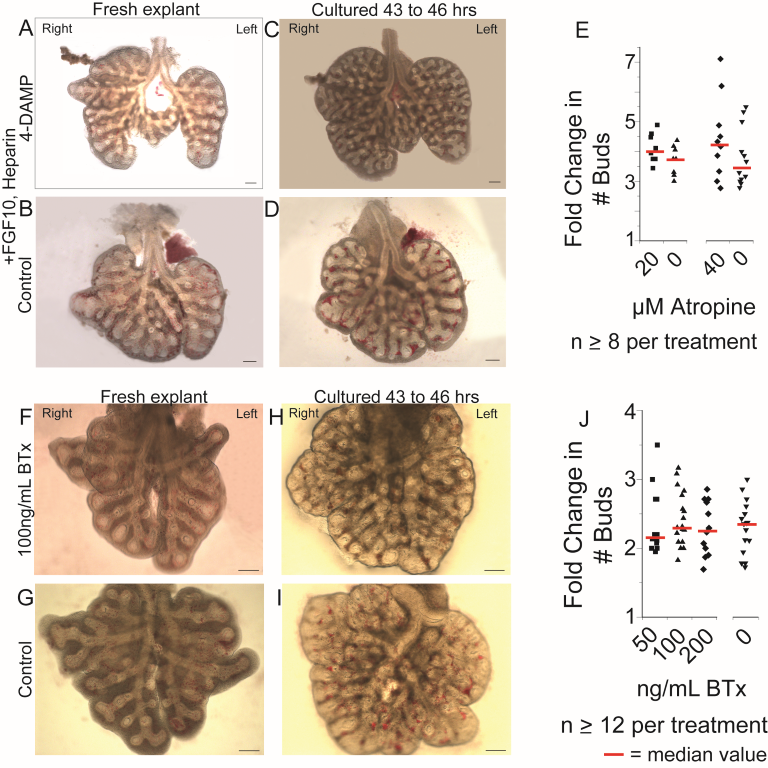

Supplement: Additional file 1: Figure S1. — Unlike the submandibular gland, inhibition of cholinergic receptors does not affect lung branching. Explants were treated with or without the irreversible muscarinic inhibitor 4-DAMP, alone or in combination with branching mediators heparin and FGF10. Freshly explanted lungs treated with 4-DAMP (A) or without inhibitor (B) are shown. The same lungs after 46 hours in culture are shown following culture with 4-DAMP (C) or without (D). At no concentration of 4-DAMP was an inhibitory effect on branching measured. The lung in (C) has more branches than the one in (D) owing to its slightly greater maturity upon explant despite coming from sibling embryos of the same pregnant mother. The branching is proportional to their starting maturity. Scale bars = 200 μm. (E) shows the results of the same experiments repeated with atropine rather than 4-DAMP. Again, neither dose of atropine impaired budding. (F-J) Lungs were cultured with botulinum toxin A starting at (F) and concluding at (H); or without the toxin, starting at (G) and seen after culture at (I). The persistence of normal budding with botulinum toxin is seen in the graph (J). [file 12915_2014_92_MOESM1_ESM.tif]
