# Supplementary material for: Airway branching has conserved needs for local parasympathetic innervation but not neurotransmission
Source: BMC Biol. 2014 Nov 11;12:92. doi: 10.1186/s12915-014-0092-2 (PMC4255442; doi:10.1186/s12915-014-0092-2)
Supplement: Additional file 2: Table S1. — Antibodies used for mammalian lung or Drosophila studies. [file 12915_2014_92_MOESM2_ESM.doc]

**Supplementary Table 1: Antibodies used for mammalian lung or *Drosophila* studies**

**DSHB=Developmental Studies Hybridoma Bank**

| **Antibody target** | **Dilution** | **Product Info** |
| --- | --- | --- |
| Β3-tubulin | 1:250 | Covance MMS-435P |
| Cleaved caspase 3 | 1:500 | R&D AF835 |
| Fasciclin II | 1:3 | DSHB 1D4 |
| eGFP | 1:1000 | Invitrogen A11122 |
| Isotype specific donkey secondary antibodies | 1:500 | Invitrogen |
| Isotype specific goat secondary antibodies | 1:500 | Invitrogen |
| Phosphohistone H3 | 1:500 | Millipore 06-570 |
| Stranded at second | 1:1000 | D. Cavener, Vanderbilt U. |
| Tracheal lumen 2A12 | 1:2 | DSHB 2A12 |
| VEGFR2 | 1:25 | BD Biosciences 560680 |
